# Endothelial Cells Can Regulate Smooth Muscle Cells in Contractile Phenotype through the miR-206/ARF6&NCX1/Exosome Axis

**DOI:** 10.1371/journal.pone.0152959

**Published:** 2016-03-31

**Authors:** Xiao Lin, Yu He, Xue Hou, Zhenming Zhang, Rui Wang, Qiong Wu

**Affiliations:** 1 MOE Key Laboratory of Bioinformatics, School of Life Sciences, Tsinghua University, Beijing 100084, China; 2 Department of basic medicine, Medical College of Qinghai University, Xining 810016, China; University of Nevada School of Medicine, UNITED STATES

## Abstract

Active interactions between endothelial cells and smooth muscle cells (SMCs) are critical to maintaining the SMC phenotype. Exosomes play an important role in intercellular communication. However, little is known about the mechanisms that regulate endothelial cells and SMCs crosstalk. We aimed to determine the mechanisms underlying the regulation of the SMC phenotype by human umbilical vein endothelial cells (HUVECs) through exosomes. We found that HUVECs overexpressing miR-206 upregulated contractile marker (α-SMA, Smoothelin and Calponin) mRNA expression in SMCs. We also found that the expression of miR-206 by HUVECs reduced exosome production by regulating ADP-Ribosylation Factor 6 (ARF6) and sodium/calcium exchanger 1 (NCX1). Using real-time PCR and western blot analysis, we showed that HUVEC-derived exosomes decreased the expression of contractile phenotype marker genes (α-SMA, Smoothelin and Calponin) in SMCs. Furthermore, a reduction of the miR-26a-containing exosomes secreted from HUVECs affects the SMC phenotype. We propose a novel mechanism in which miR-206 expression in HUVECs maintains the contractile phenotype of SMCs by suppressing exosome secretion from HUVECs, particularly miR-26a in exosomes, through targeting ARF6 and NCX1.

## Introduction

Endothelial cells are adjacent to smooth muscle cells (SMCs) in blood vessels. The interaction between endothelial cells and SMCs is important for maintaining normal vascular physiology and structure [1]. The intercellular communication between endothelial cells and SMCs could occur through direct contact or through paracrine signalling [2]. As previously reported, endothelial cells can also secrete exosomal signalling molecules to regulate SMCs [3].

Exosomes are membrane vesicles with a diameter of 30–100 nm that are secreted by various cell types [4]. Exosomes originate as multivesicular bodies (MVBs) by endosomal membrane budding into its lumen [5]. Subsequently, portions of the MVB release its internal vesicles into the extracellular space by fusing with the plasma membrane to release exosomes [6]. The role of exosomes is to transport endogenous microRNAs (miRNAs), mRNA and proteins from secretory cells to recipient cells [7, 8]. In recent years, the exosome has been shown to be involved in many intercellular behaviours. For example, vesicles secreted from KLF2-expressing endothelial cells into the extracellular space are enriched in miR-143/145 and control the SMC phenotype [3]. MiR-150 secreted from monocytes in microvesicles have been shown to promote capillary tube formation and endothelial cell angiogenesis [9]. MiR-214-enriched exosomes secreted from tumours can promote Treg expansion from T cells and, in turn, promote tumour growth [10].

Many proteins and lipids participate in exosome biogenesis and secretion [11, 12]. ADP-Ribosylation Factor 6 (ARF6) was recently identified as a regulator of intraluminal vesicles budding and exosome biogenesis [13]. ARF6 is a member of the GTP-binding protein family and localizes at the plasma membrane in association with endosomes [14]. The function of ARF6 is to regulate the cortical actin cytoskeleton, cell migration, and membrane traffic, among other processes [15]. In addition, an increase in intracellular calcium concentrations can promote exosome release in K562 cells [16].

**Fig 1:**
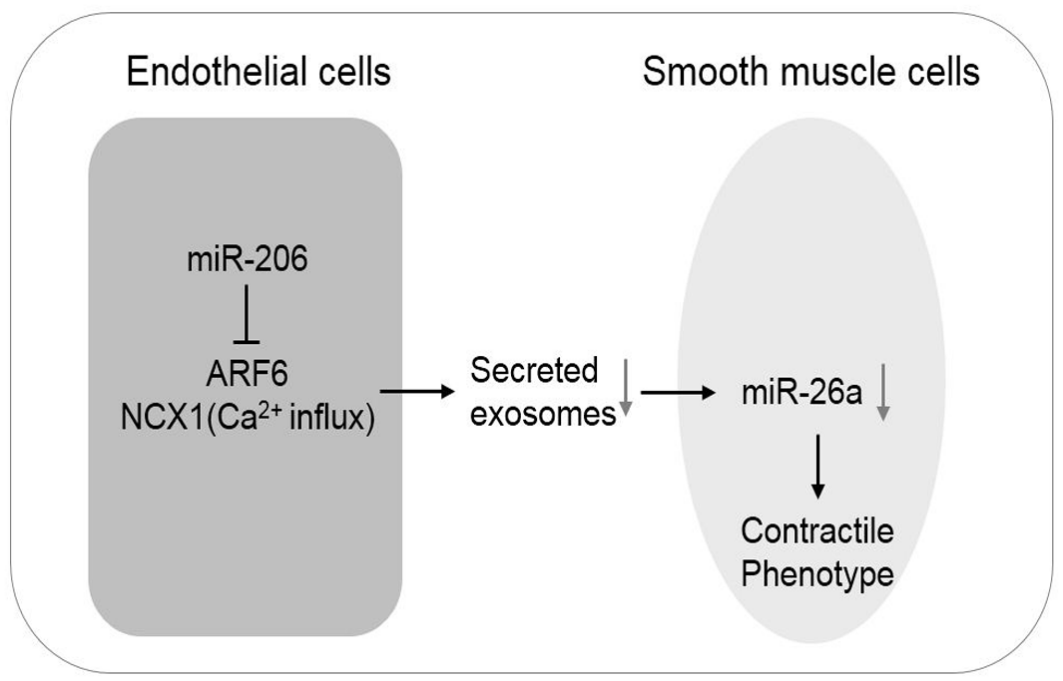
Graphical Abstract. Endothelial cells regulate the SMC phenotype by modulating the quantity of exosomes through the miR-206/ARF6 and NCX1/exosome axis.

In the current study, we investigated the effect of endothelial cells on the SMC phenotype through exosomes. We found that the ectopic expression of miR-206 in human umbilical vein endothelial cells (HUVECs) enhanced the contractile SMC phenotype in a co-culture system. miR-206 decreased exosome production in HUVECs through ARF6 and sodium/calcium exchanger 1 (NCX1). Furthermore, a decrease in exosomes and exosomal miR-26a from HUVECs promoted the SMC contractile phenotype. These data suggest that the downregulation of exosome production in HUVECs by miR-206 through ARF6 and NCX1 contributes to the maintenance of the SMC contractile phenotype (Fig 1).

## Material and Methods

### Cell Culture

Human umbilical vein endothelial cells (HUVECs) were purchased from Cyagen Biosciences (Guangdong, China) and cultured in endothelial basal medium-2 supplemented with EGM-2 (Lonza, Basel, Switzerland). Human aortic smooth muscle cells (SMCs) were purchased from LIFELINE cell technology (Frederick, MD, USA) and cultured in VascuLife Basal Medium (LIFELINE cell technology, Frederick, MD, USA). GW4869 (Sigma-Aldrich, St. Louis, MO, USA) was added to the expansion medium as needed.

### Exosome isolation

Exosomes were isolated by a multi-step centrifugation process as described previously [17]. Briefly, HUVECs were grown to 60% confluence in a T25 flask (Corning Life Science, Tewksbury, MA, USA) and transfected with 200 pmol of siRNA or miRNA. After 72 h of culture, the culture medium was collected and centrifuged at 800 g for 10 min and again at 12,000 g for 30 min at 4°C to remove cell debris, after which the supernatants were transferred to a fresh tube and centrifuged at 100,000 g for 1 h at 4°C. The pelleted vesicles were washed with ice-cold PBS and centrifuged at 100,000 g for 1 h at 4°C. Finally, the pelleted exosomes were resuspended in the appropriate medium.

### Exosome characterization

Exosome size was measured using the Zetasizer Nano ZS (Malvern Instruments Ltd., Malvern, Worcestershire, UK) according to the manufacturer's instructions. Exosome morphology was investigated using transmission electron microscopy (TEM) [18, 19]. The pellet sample was prepared for uranyl acetate negative staining. A 20 μl aliquot containing purified exosomes was dropped on a carbon-coated grid for 2 min. The grid was subsequently incubated in 2% uranyl acetate for 2 min and dried on the edge of a filter paper. The preparation was visualized by transmission electron microscopy at 80 kV using a Hitachi H-7650B microscope (Tokyo, Japan).

### Cell transfection

A total of 75 pmol miRNA, antagomiRNAs or siRNAs (Table 1) was transfected using Lipofectamine RNAi MAX Reagent (Invitrogen, Carlsbad, CA, USA), and 2.5 μg plasmid DNA was transfected using Lipofectamine 2000 (Invitrogen, Carlsbad, CA, USA) into cells cultured on a 6-well plate (Corning Life Science, Tewksbury, MA, USA). MiRNA and antagomiRNA oligos were synthesised by GenePharma Co., Ltd (Shanghai, China).

**Table 1:** siRNA Sequences.

siRNA	Sequence
siARF6	GCACCGCAUUAUCAAUGACCG
siNCX1	GGUGGUGAUUUGACUAACATT

### RNA isolation and quantitative real-time PCR

Total RNA was isolated from HUVECs and SMCs using the miRcute miRNA Isolation Kit (Tiangen, Beijing, China). Exosomal RNAs were isolated using the total exosome RNA and protein isolation kit (Invitrogen, Carlsbad, CA, USA). The Agilent 2100 Bioanalyzer (Agilent Technologies, Santa Clara, CA, USA) was used to analyze the total cell RNA and total exosomal RNA. mRNA analysis was performed using the SuperReal PreMix kit (SYBR Green) (Tiangen, Beijing, China), and miRNA analysis was performed using miRcute miRNA qPCR detection Kit (Tiangen, Beijing, China). Primer sequences are listed in Table 2. U6 and GAPDH were used as endogenous controls for miRNA and mRNA detection, respectively.

**Table 2:** Real-time PCR primers.

Gene	Forward Primer 5' to 3'	Reverse Primer 5' to 3'
GAPDH	GGAGCGAGATCCCTCCAAAAT	GGCTGTTGTCATACTTCTCATGG
ARF6	ATGGGGAAGGTGCTATCCAAAATCT	CCGCCCACATCCCATAC
NCX1	GCGATTGCTTGTCTCGGGTC	CCACAGGTGTCCTCAAAGTCC
SRF	CGAGATGGAGATCGGTATGGT	GGGTCTTCTTACCCGGCTTG
a-SMA	GTGATGGTGGGAATGGG	CAGGGTGGGATGCTCTT
Calponin	CACTGAGCAACGCTATTCCA	AACGCCACTGTCACATCCAC
Smoothelin	GGCTCTGTGCGGGATCGTGT	CCTCGTTGCTCCTTGCTGAA
SM22a	CGGCAGATCATCAGTTAGAGC	GCCCAGGTGCAGTTACCA

### Western blot analysis

Whole-cell lysates extracted using RIPA (Radio Immunoprecipitation Assay) Lysis Buffer (Huaxingbio Biotechnology, Beijing, China), and exosome fractions in PBS were solubilized in SDS-PAGE loading buffer and heated (95°C, 5 min) for western blot. Antibodies against Hsp70 and CD63 were purchased from Santa Cruz Biotechnology (Santa Cruz, CA, USA), anti-ARF6, NCX1, α-SMA and Calponin antibodies were obtained from Abcam (Cambridge, UK), anti-SRF antibodies were obtained from CST Inc. (Danvers, MA, USA). SDS-PAGE was performed using 12% Tris-Glycine Gels (Huaxingbio, Beijing, China), and the protein was transferred to Polyvinylidene Difluoride (PVDF) membranes (Millipore Corporation, Bedford, MA, USA). The membranes were blocked in 5% nonfat dried milk powder (Huaxingbio Biotechnology, Beijing, China) in TBST for 1 h at room temperature and incubated with primary antibodies overnight at 4°C. Secondary antibodies conjugated with horseradish peroxidase (HRP, Sigma-Aldrich, St. Louis, MO, USA) were incubated with the membranes for 2 h. The blots were visualized by ECL chemiluminescence reagents from Pierce Biotechnology (Rockford, IL, USA).

### Co-culture experiments

Cell co-cultures were performed using a transwell system. Primary cultured SMCs (1×10^5^ cells/well) in the bottom compartment were washed twice with PBS before treatment with transfected HUVECs or purified exosomes. Subsequently, the transfected HUVECs (1×10^5^ cells) or purified exosomes alone were added to the top compartment of the transwell system. The SMCs were cultured for an additional 72 h and collected for the extraction of total RNA or total protein.

SMCs were co-cultured with HUVECs transfected with cel-miR-39 for 1, 4, 8 or 24 h. The N-SMase inhibitor GW4869 (5 μM) was added to HUVECs transfected with cel-miR-39 as described previously [3] and incubated for 24 h. Total RNA was extracted for further analysis.

### Dual-luciferase assay

The wild-type and mutant (C to A mutation of target sites) 3’UTR fragments of target genes were cloned into the pGL3 luciferase reporter vector (Qinglan Bio, Suzhou, China). Aliquots containing 100 ng of each reporter vector (pGL3-ARF6, pGL3-ARF6-mutant, pGL3-NCX1 and pGL3-NCX1-mutant) were co-transfected with 5 pmol of miRNA or antagomiRNA into HeLa cells in a 96-well plate (Corning Life Science, Tewksbury, MA, USA), and the cells were cultured for 24 h. Luciferase activity was measured using the Dual-Luciferase^®^ Reporter Assay System (Promega BioSciences, LLC., San Luis Obispo, CA, USA).

### DHPE labelling of MVBs

MVBs were labelled with rhodamine-DHPE (Rhodamine B 1,2-Dihexadecanoyl-*sn*-Glycero-3-Phosphoethanolamine, Invitrogen, Carlsbad, CA, USA) as described previously [17]. Briefly, 1 μl of DHPE stock solution (5 mg/ml) was diluted in 50 μl chloroform and solubilized in absolute ethanol. The ethanolic solution was injected into serum-free EBM-2 under vigorous vortexing. The mixture was added to cells seeded onto glass-bottom dishes (MatTek, Ashland, MA, USA), and incubated for 30 min at 37°C. The medium was removed, and the cells were washed twice with ice-cold PBS. Cells were subsequently cultured in complete EBM-2 medium (Lonza, Basel, Switzerland) for 4–8 h before analysis by FV10i-Oil confocal fluorescence microscopy (Olympus Corporation, Tokyo, Japan).

### Detection of intracellular calcium with Fluo-3AM

Cell monolayers were treated with 0.5 μM Fluo-3AM and incubated for 1 h at 37°C. The medium was removed, and the cells were washed twice in ice-cold PBS. Complete EBM-2 medium (Lonza, Basel, Switzerland) was subsequently added, and the cells were incubated for an additional 20–30 min at 37°C. The cells were analyzed by FV10i-Oil confocal fluorescence microscopy (Olympus Corporation, Tokyo, Japan).

### Actin cytoskeleton staining

SMCs (1×10^5^ cells/well) were seeded onto glass-bottom dishes (MatTek, Ashland, MA, USA) and cultured in the presence or absence of exosomes for 72 h. The SMCs were washed twice with ice-cold PBS, fixed in 4% paraformaldehyde in PBS for 15 min, and washed twice with ice-cold PBS after fixation. SMCs were permeabilized with 0.5% Triton X-100 in PBS for 10 min and washed three times in ice-cold PBS. The cells were stained with 0.5 μg/ml Phalloidin-TRITC (Sigma-Aldrich, St. Louis, MO, USA) for 40 min and washed twice in ice-cold PBS. Finally, the cells were stained with 1 μg/ml DAPI (4,6-diamidino-2-phenylindole, Huaxingbio Biotechnology, Beijing, China) for 3 min and washed twice in ice-cold PBS. The cells were analyzed by FV1200 confocal fluorescence microscopy (Olympus Corporation, Tokyo, Japan).

### Statistical analysis

Data were expressed as the mean (n ≥ 3) ± standard deviation (SD). Student’s *t*-test was used for experiments comparing two groups, and an ANOVA was used for comparison of more than two independent groups. p values corresponding to *p*<0.01 or *p*<0.05 were considered statistically significant and are indicated by one or two stars, respectively.

## Results

### Ectopic miR-206 expression in HUVECs induces the SMC contractile phenotype

**Fig 2:**
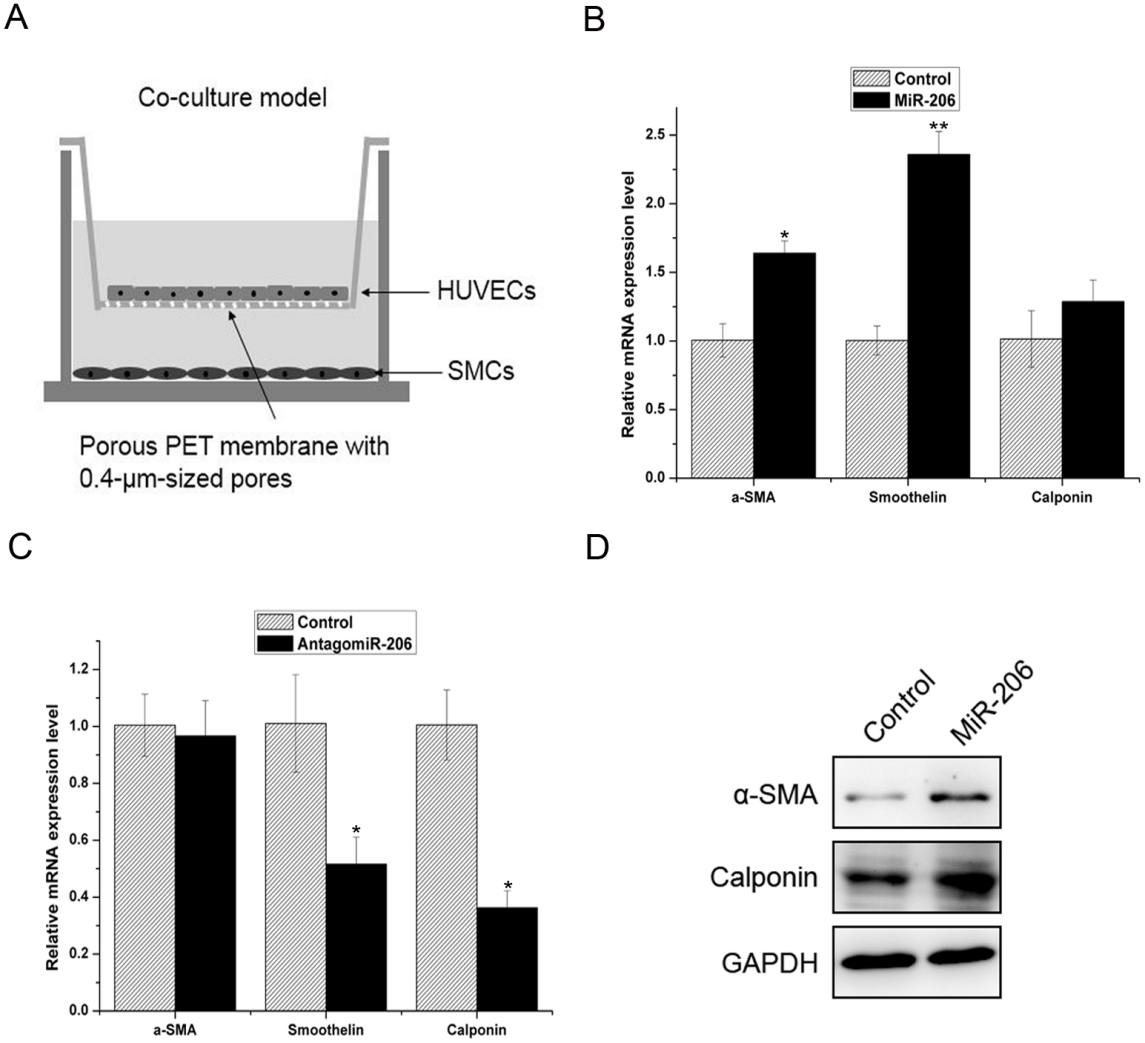
Ectopic miR-206 expression in HUVECs induces the SMC contractile phenotype. (A) An *in vitro* transwell system was used for co-culture experiments. HUVECs were seeded in the top compartment and SMCs were cultured in the bottom compartment. Exosomes mediated the regulation of SMCs by HUVECs. (B) Real-time PCR analysis of contractile marker gene expression (α-SMA, Smoothelin and Calponin) in SMCs co-cultured with miR-206-overexpressing HUVECs. (C) Real-time PCR analysis of contractile marker gene expression (α-SMA, Smoothelin and Calponin) in SMCs co-cultured with antagomiR-206-overexpressing HUVECs. (D) Western blot analysis of contractile marker gene expression (α-SMA and Calponin) in SMCs co-cultured with miR-206-overexpressing HUVECs. Three independent experiments were performed for each condition, and data are presented as the mean ± SEM. * *p*<0.05 and ** *p*<0.01 versus control group.

It has been reported that the human serum exosomal-miR-206 levels immediately increased and were stable for 24 h following participation in a marathon [20]. Serum exosomal-miRNA was taken up by endothelial cells and may have a regulatory role [21]. To determine whether miR-206 in HUVECs was involved in regulating SMC phenotype transformation, we used the transwell system to study the regulative effect of mock- and miR-206-overexpressing HUVECs on the SMC phenotype. This system prevented cells from direct contact by separating them using a membrane with a 0.4 μm pore size (Fig 2A). SMCs were seeded in the lower compartment, and HUVECs were seeded in the upper compartment after transfection for 72 h. After 72 h, we measured the expression of contractile marker genes (α-SMA, Smoothelin and Calponin) in SMCs by real-time PCR and western blot. The results showed that HUVECs overexpressing miR-206 upregulated contractile marker mRNA expression in SMCs (Fig 2B). In contrast, the inhibition of miR-206 in HUVECs downregulated the mRNA expression of contractile marker genes in SMCs (Fig 2C). The western blot results showed that HUVECs overexpressing miR-206 also upregulated α-SMA and Calponin protein expression (Fig 2D). These results suggest that miR-206 expression in HUVECs contributed to the SMC contractile phenotype.

### MiR-206 post-transcriptionally controls ARF6 and NCX1 expression

**Fig 3:**
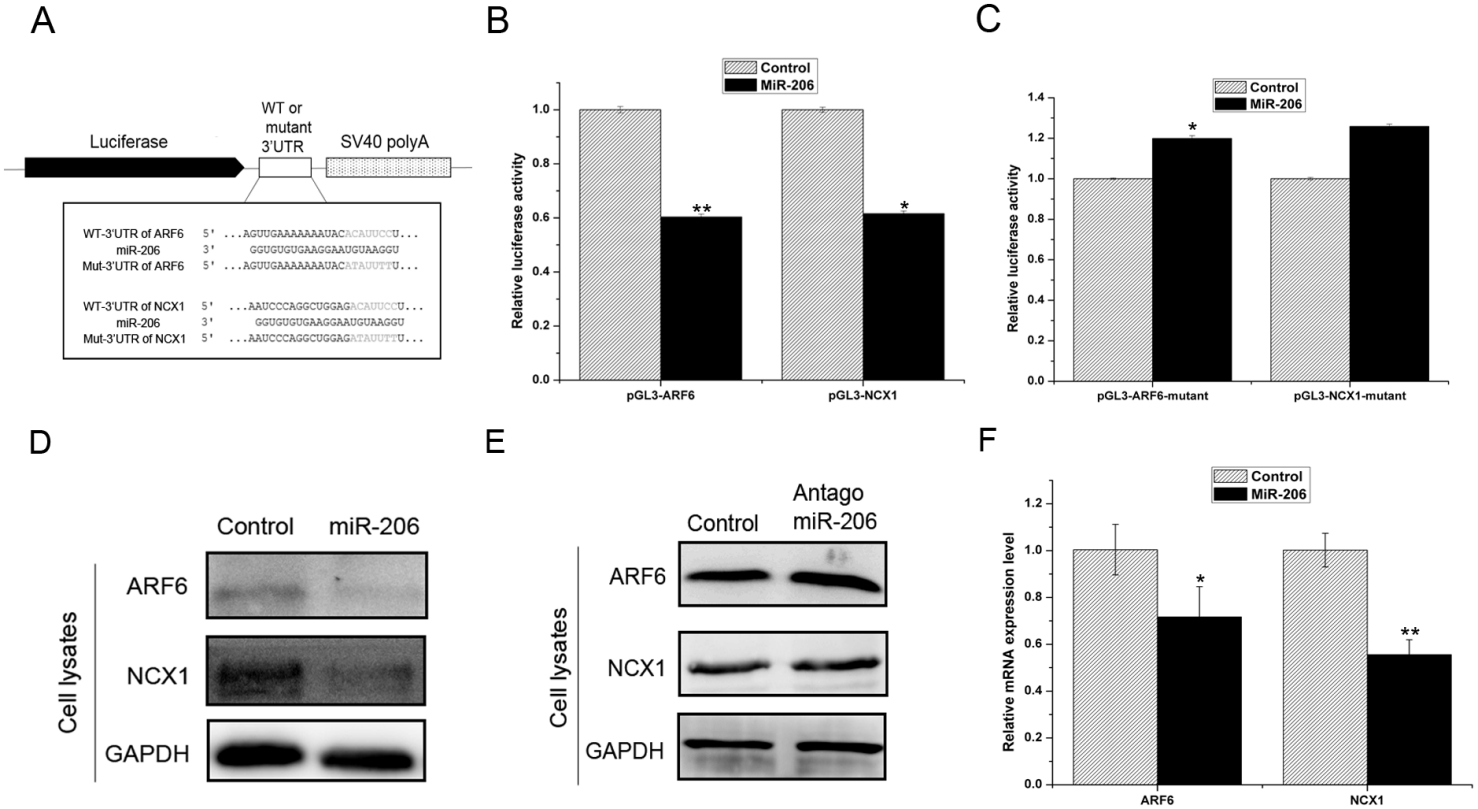
MiR-206 post-transcriptionally controls ARF6 and NCX1 expression. (A) Illustration of the construction of pGL3 vectors containing either the wild-type or mutant 3’UTR of ARF6 and NCX1. (B, C) Measurement of luciferase activity in HeLa cells. (D, E) Western blot analysis of ARF6 and NCX1 protein expression in HUVECs transfected with miR-206 (D) or antagomiR-206 (E). (F) Real-time PCR analysis of ARF6 and NCX1 expression in HUVECs. Three independent experiments were performed for each condition, and the data are presented as the mean ± SEM. * *p*<0.05 and ** *p*<0.01 versus control group.

Bioinformatics analysis with Pictar, Targetscan and miRanda predicted that miR-206 targets the 3’UTRs of ARF6 and NCX1 (in grey) (Fig 3A). To confirm that miR-206 directly targeted ARF6 and NCX1, we introduced wild-type or mutated ARF6 and NCX1 3’UTRs into the pGL3 luciferase reporter vector. We examined miR-206 binding to the ARF6 and NCX1 3’UTRs using a dual-luciferase reporter assay in HeLa cells. We co-transfected miR-206 with the wild-type 3’UTRs of pGL3-ARF6 or pGL3-NCX1 into HeLa cells and allowed the cells to incubate for 24 h. The dual-luciferase assay results showed that miR-206 repressed the luciferase activities of wild-type pGL3-ARF6 and pGL3-NCX1 (Fig 3B). We confirmed the specificity of miR-206 binding to the 3’UTRs of ARF6 and NCX1 by performing the same reporter assay using a constructs containing a mutated miR-206 seed sequence in the 3’UTRs of ARF6 or NCX1. The results showed that mutations in the 3’UTRs of pGL3-ARF6-mutant and pGL3-NCX1-mutant each abolished the repression of luciferase activity (Fig 3C). These data indicate that miR-206 directly targets the 3’UTRs of ARF6 and NCX1.

We next examined the effects of miR-206 on ARF6 and NCX1 protein expression by transfecting miR-206 and antagomiR-206 into HUVECs. The western blot results confirmed that miR-206 inhibited both ARF6 and NCX1 protein expression (Fig 3D and 3E). The real-time PCR results also showed that miR-206 inhibited ARF6 and NCX1 expression (Fig 3F). Together, these data suggest that miR-206 regulates ARF6 and NCX1 expression at the post-transcriptional level.

### ARF6 and NCX1 mediate exosome production in HUVECs

**Fig 4:**
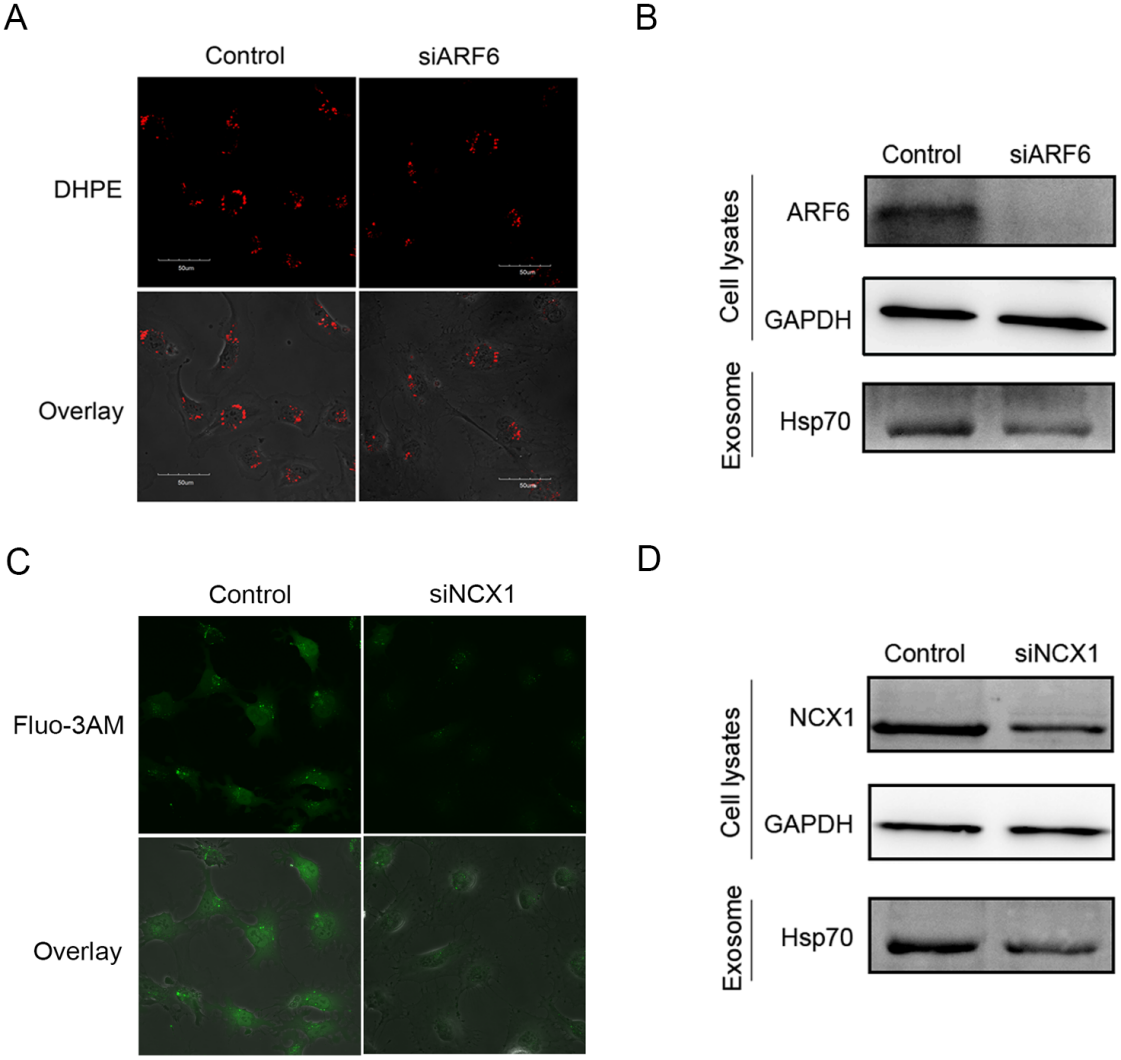
ARF6 and NCX1 mediate exosome production in HUVECs. (A) DHPE staining of intracellular MVBs assayed by confocal microscopy at 600× magnification. (B) Western blot analysis of Hsp70 protein expression in exosomes secreted from ARF6 knockdown HUVECs. (C) Fluo-3AM staining of intracellular calcium assayed by confocal microscopy at 600× magnification. (D) Western blot analysis of Hsp70 protein expression in exosomes secreted from NCX1 knockdown HUVECs.

ARF6 has been reported to control exosome biogenesis in MCF-7 cells [13], and increased intracellular calcium can promote exosome secretion in K562 cells [16]. Thus, we hypothesized that ARF6 could impact exosome production in HUVECs. We knocked down ARF6 expression in HUVECs using siARF6 and stained intracellular MVBs using DHPE. The staining results showed that intracellular exosome biogenesis was indeed inhibited by siARF6 (Fig 4A). To determine whether ARF6 affected extracellular exosome production in HUVECs, we isolated exosomes from the supernatants of mock- and siARF6-transfected HUVECs 72 h post-transfection. We analyzed exosome production by western blot. The results showed that siARF6 reduced Hsp70 protein expression (Fig 4B). Because NCX1 is a bi-directional calcium balancer in several cell types, we investigated whether NCX1 affected the intracellular calcium concentration in HUVECs. We transiently transfected HUVECs with mock or siNCX1 and cultured for 24 h and then measured intracellular calcium concentrations by Fluo-3AM staining. NCX1 knockdown reduced the intracellular calcium concentration (Fig 4C), indicating that NCX1 acts as a calcium influx protein in HUVECs. Subsequently, we analyzed the role of NCX1 in regulating exosome secretion. We isolated exosomes from the supernatants of mock- and siNCX1-transfected HUVECs 72 h post-transfection. The western blot results showed that siNCX1 reduced Hsp70 protein expression (Fig 4D), suggesting that NCX1 promoted exosome secretion in HUVECs.

### MiR-206 reduces exosome production in HUVECs

**Fig 5:**
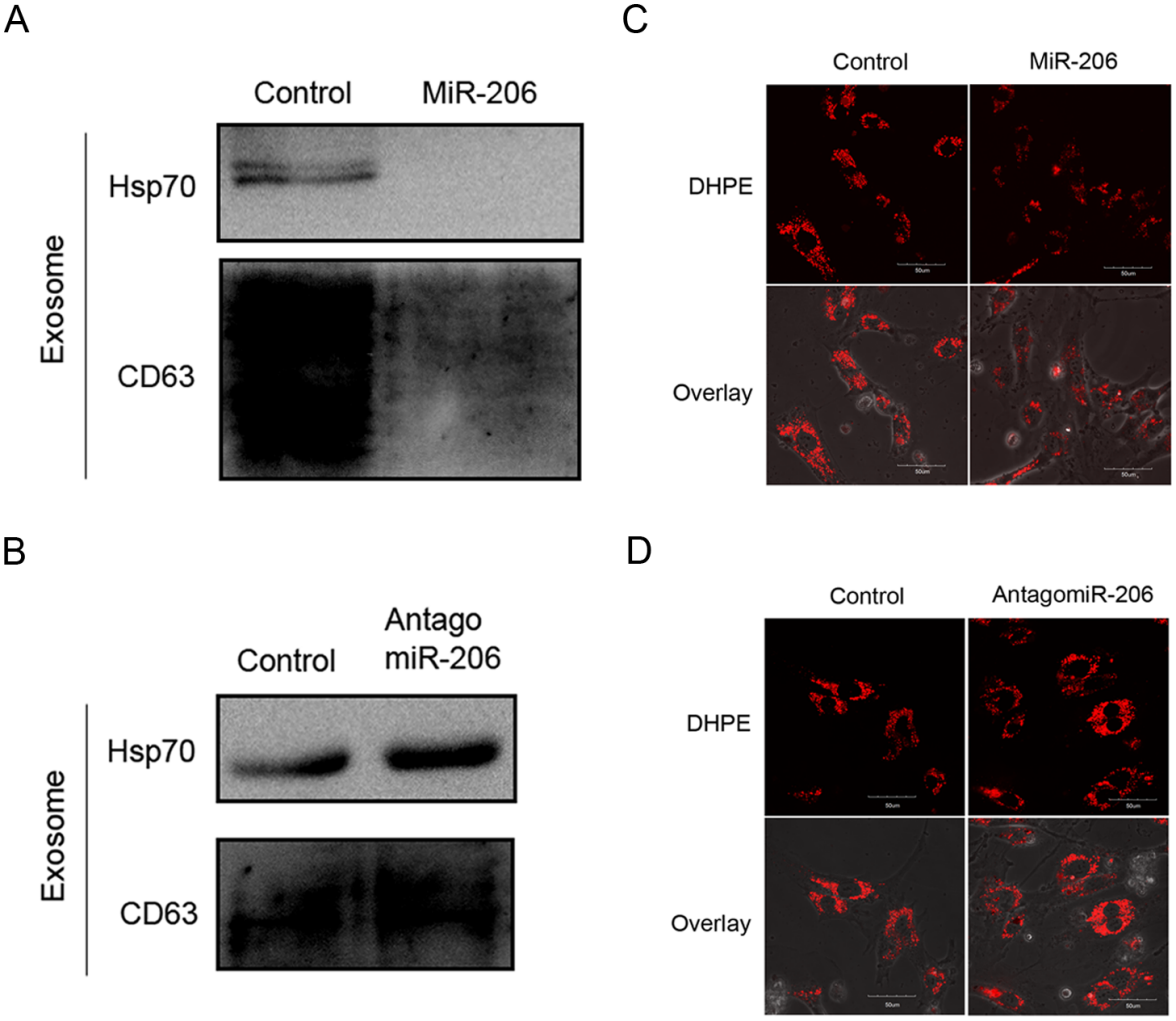
MiR-206 reduces HUVEC exosome production. (A, B) Western blot analysis of Hsp70 and CD63 expression in secreted exosomes from HUVECs transiently transfected with miR-206 (A) or antagomiR-206 (B). (C, D) DHPE staining of intracellular MVBs in HUVECs assayed by confocal microscopy at 600× magnification.

Because miR-206 could target ARF6 and NCX1, which are both involved in exosome production, we hypothesized that miR-206 influenced the SMC phenotype by reducing the exosome production in HUVECs. To study the effect of miR-206 on exosome production in HUVECs, we transiently transfected cells with miR-206 or antagomiR-206. We isolated extracellularly secreted exosomes 72 h post-transfection and analyzed exosome production by western blot analysis. The western blot results showed that miR-206 inhibited the expression of the exosome markers CD63 and Hsp70 (Fig 5A and 5B). In addition, we measured intracellular MVBs by DHPE staining 24 h after miR-206 or antagomiR-206 transfection into HUVECs. DHPE staining showed that miR-206 transfection decreased the number of intracellular MVBs in HUVECs (Fig 5C and 5D). These data indicate that miR-206 reduced exosome production in HUVECs.

### HUVEC exosomes attenuate the SMC contractile phenotype

**Fig 6:**
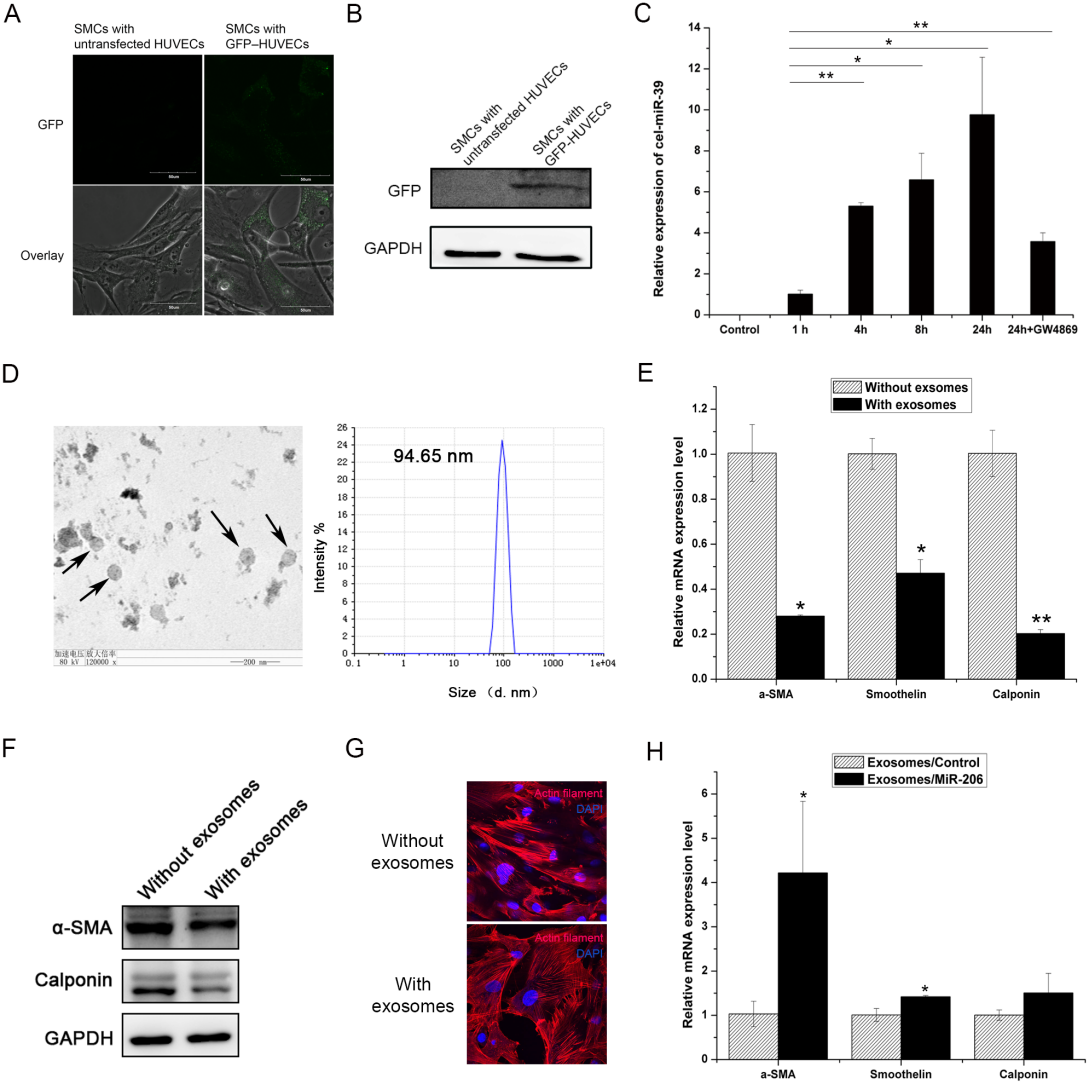
HUVEC exosomes attenuate the SMC contractile phenotype. (A) GFP expression in SMCs visualized by confocal microscopy at 600× magnification. (B) Western blot analysis of GFP protein in SMCs. (C) Real-time PCR analysis of cel-miR-39 expression in SMCs. (D) Determination of exosome size using a Zetasizer Nano instrument and transmission electron microscopy (indicated by black arrow). (E) Real-time PCR analysis of contractile marker gene expression (α-SMA, Smoothelin and Calponin) in SMCs in the presence or absence of exosomes. (F) Western blot analysis of contractile marker gene expression (α-SMA and Calponin) in SMCs in the presence or absence of exosomes. (G) Confocal microscopy of intracellular actin filament organization of SMCs by staining with Phalloidin-TRITC at 600× magnification. (H) Real-time PCR analysis of phenotype marker gene expression (α-SMA, Smoothelin and Calponin) in SMCs treated with exosomes from miR-206-overexpressing HUVECs. Three independent experiments were performed for each condition, and the data are presented as the mean ± SEM. * *p*<0.05 and ** *p*<0.01 versus control group.

It has been previously shown that exosomes function as extracellular vesicles in intercellular communication [3, 19]. We examined whether exosomes are involved in the protein and miRNA-mediated communication between HUVECs and SMCs. We used GFP expression as a marker to measure exosome transmission from HUVECs to SMCs. We transduced HUVECs with a GFP expression cassette using a lentiviral vector and co-cultured them with SMCs in a transwell dish. After 72 h of co-culture, we analyzed GFP expression in SMCs by confocal microscopy and western blot analysis. The results showed that GFP was indeed transferred from HUVECs to SMCs during co-culture (Fig 6A and 6B). Additionally, we transfected HUVECs with cel-miR-39, which is a *Caenorhabditis elegans* miRNA. We analyzed the expression of cel-miR-39 in SMCs after co-culture by real-time PCR and found that cel-miR-39 expression was upregulated in SMCs, indicating that miRNAs could be transferred from HUVECs to SMCs (Fig 6C). Furthermore, the inhibition of exosome generation using a sphingomyelinase inhibitor (GW4869) [3, 22] attenuated the transfer of cel-miR-39 to SMCs, further suggesting that exosomes mediated the transfer of miRNAs from HUVECs to SMCs (Fig 6C).

To test whether HUVEC-derived exosomes could affect the SMC phenotype, we cultured SMCs for 72 h in the presence or absence of exosomes purified from HUVEC cultures. We measured exosome size using a Zetasizer Nano instrument and transmission electron microscopy and found the average diameter to be approximately 95 nm, which is consistent with previous reports [4, 23] (Fig 6D). Additionally, the real-time PCR results showed that compared to normal SMCs, the expression of contractile marker genes (α-SMA, Smoothelin and Calponin) was significantly decreased in SMCs cultured with exosomes (Fig 6E). Western blot showed that α-SMA and Calponin protein expression also decreased (Fig 6F). Consequently, we followed the intracellular actin filament organization of SMCs cultured in the presence or absence of exosomes using Phalloidin-TRITC. SMCs cultured with exosomes showed disorganized actin filaments and lost their actin organization (Fig 6G).

To confirm that exosomes from miR-206-overexpressing HUVECs were modulating the SMC phenotype, we treated SMCs with purified original exosomes, which were secreted from a similar number of mock- or miR-206-overexpressing HUVECs. Real-time PCR showed that the exosomes from miR-206-overexpressing HUVECs upregulated the expression of contractile marker genes (α-SMA, Smoothelin and Calponin) in SMCs (Fig 6H). Taken together, the results indicate that the reduced number of exosomes from miR-206-overexpressing HUVECs accounts for the regulation of the SMC phenotype.

### MiR-26a mediates the regulation of SMCs by miR-206-overexpressing HUVECs

**Fig 7:**
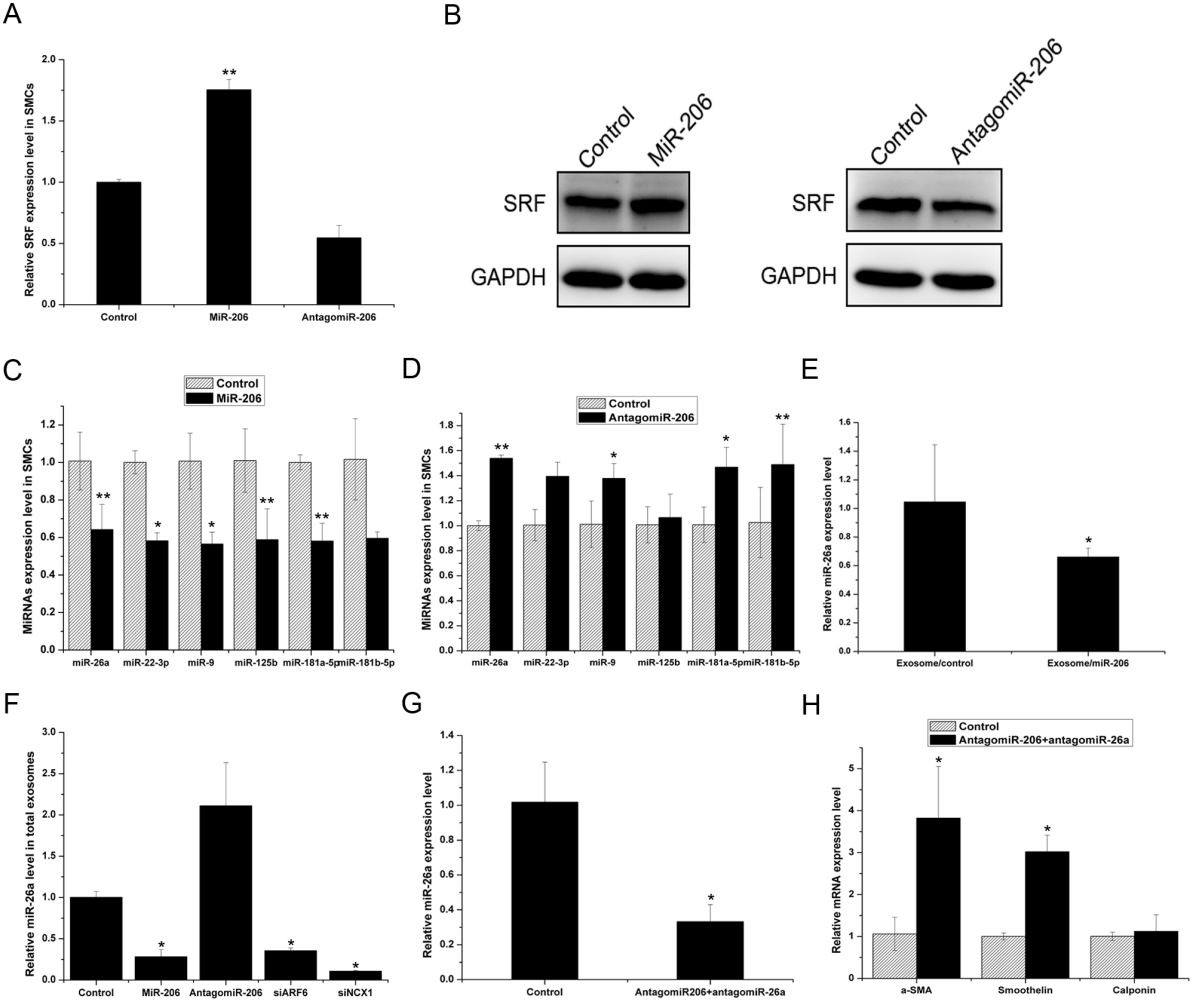
MiR-26a mediates the SMC phenotype regulation co-cultured with miR-206-overexpressing HUVECs. (A) Real-time PCR analysis of SRF expression in SMCs. (B) Western blot analysis of SRF protein level in SMCs. (C, D) Real-time PCR analysis of phenotype-related miRNA expression in SMCs co-cultured with miR-206-overexpressing HUVECs (C) and antagomiR-206-overexpressing HUVECs (D). (E) Real-time PCR analysis of miR-26a expression in SMCs treated with exosomes from miR-206-overexpressing HUVECs. (F) Real-time PCR analysis of miR-26a expression in the total exosomes from the same amounts of mock-, miR-206, antagomiR-206, siARF6 and siNCX1-transduced HUVECs. (G, H) Real-time PCR analysis of miR-26a expression (G) and phenotype marker gene expression (H) in SMCs co-cultured with HUVECs co-transfected with antagomiR-26a and antagomiR-206 or mock. Three independent experiments were performed for each condition, and data are presented as the mean ± SEM. * *p*<0.05 and ** *p*<0.01 versus control group.

To assess whether SMC phenotype-related miRNAs changed in co-cultured SMCs, we evaluated the expression levels of a group of representative miRNAs. SRF is a critical positive regulator of SMC differentiation [24]. miR-206 overexpression in HUVECs significantly increased the SRF mRNA and protein levels in SMCs, and the inhibition of miR-206 in HUVECs significantly reduced the SRF mRNA and protein levels in SMCs (Fig 7A and 7B). We previously demonstrated that miR-22-3p, miR-181a-5p, miR-181b-5p, miR-125b and miR-9 could all target the SRF 3’UTR and cooperatively attenuate the contractile phenotype of SMCs (S1 Fig). Conversely, miR-26a negatively regulates SMC differentiation by targeting SMAD1 [25]. We measured the levels of these miRNAs in co-cultured SMCs by real-time PCR. The levels of miR-26a and miRNAs upstream of SRF decreased in SMCs co-cultured with miR-206-overexpressing HUVECs (Fig 7C) but increased in SMCs co-cultured with antagomiR-206-overexpressing HUVECs (Fig 7D).

Because miR-26a has been reported to be an important regulator of SMC biology, its expression level unsurprisingly changed in co-cultured SMCs. Because the miR-26a levels decreased in SMCs by exosomes secreted from miR-206-overexpressing HUVECs (Fig 7E), we investigated whether the reduced exosome production could also affect the level of miR-26a secretion via exosomes. We isolated exosomes from the supernatants of mock-, miR-206, antagomiR-206, siARF6 and siNCX1-transduced HUVECs. Real-time PCR revealed that the secreted miR-26a level in total exosomes from HUVECs significantly decreased upon the overexpression of miR-206, siARF6, siNCX1 in HUVECs and, conversely, increased upon the overexpression of antagomiR-206 (Fig 7F). Furthermore, miR-26a expression in HUVECs did not change (S2 Fig). These results are consistent with the hypothesis that reduced exosome production rather than reduced miR-26a levels in HUVECs caused the change in the secreted miR-26a levels.

We further examined whether miR-26a could mediate the effect of miR-206-overexpressing HUVECs on SMC phenotype regulation. We transfected HUVECs with both antagomiR-26a and antagomiR-206 or mock and analyzed miR-26a expression and representative phenotype marker genes in SMCs after 72 h of co-culture. The real-time PCR results showed that miR-26a expression was reduced in SMCs (Fig 7G). Additionally, contractile marker gene expression (α-SMA, Smoothelin and Calponin) was upregulated compared to the negative control (Fig 7H). Taken together with the previous observation that antagomiR-206-transfected HUVECs led to a synthetic SMC phenotype (Fig 1C), these results suggest that miR-26a affected the SMC phenotype by acting as an important downstream miR-206 target to regulate the SMC phenotype.

## Discussion

Accumulating evidence has demonstrated that exosomes play critical roles in intercellular communication. Here, we report that endothelial cells regulate the smooth muscle cell phenotype through exosomes. We performed several experiments that support our hypothesis that endothelial cells regulate the smooth muscle cell phenotype through the miR-206/ARF6 and NCX1/exosome axis.

It has been reported that the serum miR-206 levels immediately increased and were stable for 24 h after running a marathon [20]. Exosomal miRNA in serum could furthermore be taken up by endothelial cells to perform a regulatory role [21]. Because the interaction between endothelial cells and smooth muscle cells is important for maintaining normal vascular physiological structure [1], we used a co-culture system to investigate the effect of miR-206-overexpressing HUVECs on SMC phenotype regulation. Our data demonstrated that miR-206-overexpressing HUVECs indirectly upregulated contractile phenotype marker gene (a-SMA, Smoothelin and Calponin) expression in SMCs.

Exosomes play critical roles in cell-cell communication [26] and have been reported to be regulated by many elements, such as glucose starvation [27] and serotonin [28]. We found that miR-206 inhibited the expression of exosome markers CD63 and Hsp70. Furthermore, we found that miR-206 reduced exosome production through two targets: ARF6 and NCX1. ARF6 is a ubiquitously expressed gene with an important role in exosome biogenesis, and it was recently identified as a regulator of ILV budding and exosome production in the syntenin-ALIX exosome pathway [13]. Roucourt *et al*. reported that heparanase can activate the syntenin-ALIX exosome pathway [29]. It is possible that these regulators work together to control exosome biogenesis [30]. However, we found that the effect of ARF6 on exosome biogenesis was stronger than that of heparanase. Moreover, an increase in intracellular calcium concentration promotes exosome secretion [16]. NCX1 is a bi-directional calcium balancer and is broadly expressed in various cell types [31]. In the cardiovascular system, NCX1 is a well-characterized cardiac promoter that has many important roles in cardiovascular function [32, 33, 34], and the calcium transport function of NCX1 can be disrupted by miRNAs that directly downregulate its expression [35]. Therefore, because umbilical vein endothelial cells are part of the cardiovascular system, we suspected that the NCX1 gene may be involved in intracellular calcium regulation in HUVECs. We demonstrated that ARF6 and NCX1 knockdown decreased exosome production in HUVECs. Using multifaceted experimental approaches, we found that miR-206 directly targets the 3’UTRs of ARF6 and NCX1 and attenuates ARF6 and NCX1 protein and mRNA expression. Thus, this is the first report that exosomes are regulated by miRNA through two known exosome regulators: ARF6 and NCX1.

We then wanted to determine whether HUVEC-derived exosomes play a role in SMC phenotype regulation. We investigated the effect of HUVEC-derived exosomes on SMCs. We found that exosomes from HUVECs attenuated the SMC contractile phenotype, suggesting that exosome release from HUVECs directly regulated the SMC phenotype. Furthermore, purified exosomes secreted from miR-206-overexpressing HUVECs upregulated contractile marker gene (α-SMA, Smoothelin and Calponin) expression in SMCs. Thus, the reduced exosome production from miR-206-overexpressing HUVECs accounts for SMC phenotype regulation.

Accumulating evidence supports the idea that exosomal miRNAs, such as miR-214, miR-133b and miR-150, regulate the behaviour of recipient cells through exosomes [2, 19, 9]. MiR-26a is an important negative regulator of SMC differentiation, as it targets SMAD1 through the TGF-β signalling pathway. Its interactions are enriched for putative targets likely related to SMC biology [25]. In this study, we found that miR-26a levels decreased in SMCs co-cultured with miR-206-overexpressing HUVECs. Furthermore, miR-26a mediated the effect of miR-206-overexpressing HUVECs on the SMC phenotype through exosomes and caused a decrease in the level of miR-26a found in total exosomes. We also found that the inhibition of both miR-26a and miR-206 in HUVECs promoted the expression of contractile marker genes in co-cultured SMCs. These results suggest that miR-26a is more important than miR-206 itself for regulating the SMC phenotype when less material is transferred from HUVECs to SMCs through exosomes.

In summary, our results reveal that endothelial cells regulate the SMC phenotype by modulating the quantity of exosomes. MiR-206 functions as a regulator of exosome production in HUVECs by targeting the 3’UTRs of ARF6 and NCX1, which further affects the transport of exosomal miR-26a from HUVECs to SMCs. These findings highlight a new route in which endothelial cells regulate the SMC phenotype by modulating the quantity of exosomes in HUVEC-SMC communication.

## Supporting Information

S1 Fig miRNAs upstream of SRF cooperatively attenuated the SMC contractile phenotype. (A) Real-time PCR analysis of contractile marker gene expression (SM22α, α-SMA, and Smoothelin) in SMCs. (B) SRF-WT and SRF-mutant vectors co-transfected with five predicted miRNA oligos into HeLa cells. (C, D) Measurement of luciferase activity in HeLa cells. (E, F) Western blot analysis of SRF expression in SMCs co-transfected with five miRNAs (E) or antagomiRNAs (F). (G, H) Real-time PCR analysis of SRF expression in SMCs co-transfected with five miRNAs (G) or antagomiRNAs (H). Three independent experiments were performed for each condition, and data are presented as the mean ± SEM. * *p*<0.05 and ** *p*<0.01 versus control group.
(TIF)

S2 Fig miR-26a expression levels in HUVECs. (A, B, C, D) Real-time PCR analysis of miR-26a expression in HUVECs transfected with miR-206 (A), antagomiR-206 (B), siARF6 (C) or siNCX1 (D). Three independent experiments were performed for each condition, and data are presented as the mean ± SEM. * *p*<0.05 and ** *p*<0.01 versus control group.
(TIF)
