# Supplementary figures and images for: Endothelial Cells Can Regulate Smooth Muscle Cells in Contractile Phenotype through the miR-206/ARF6&NCX1/Exosome Axis
Source: PLoS One. 2016 Mar 31;11(3):e0152959. doi: 10.1371/journal.pone.0152959 (PMC4816502; doi:10.1371/journal.pone.0152959)

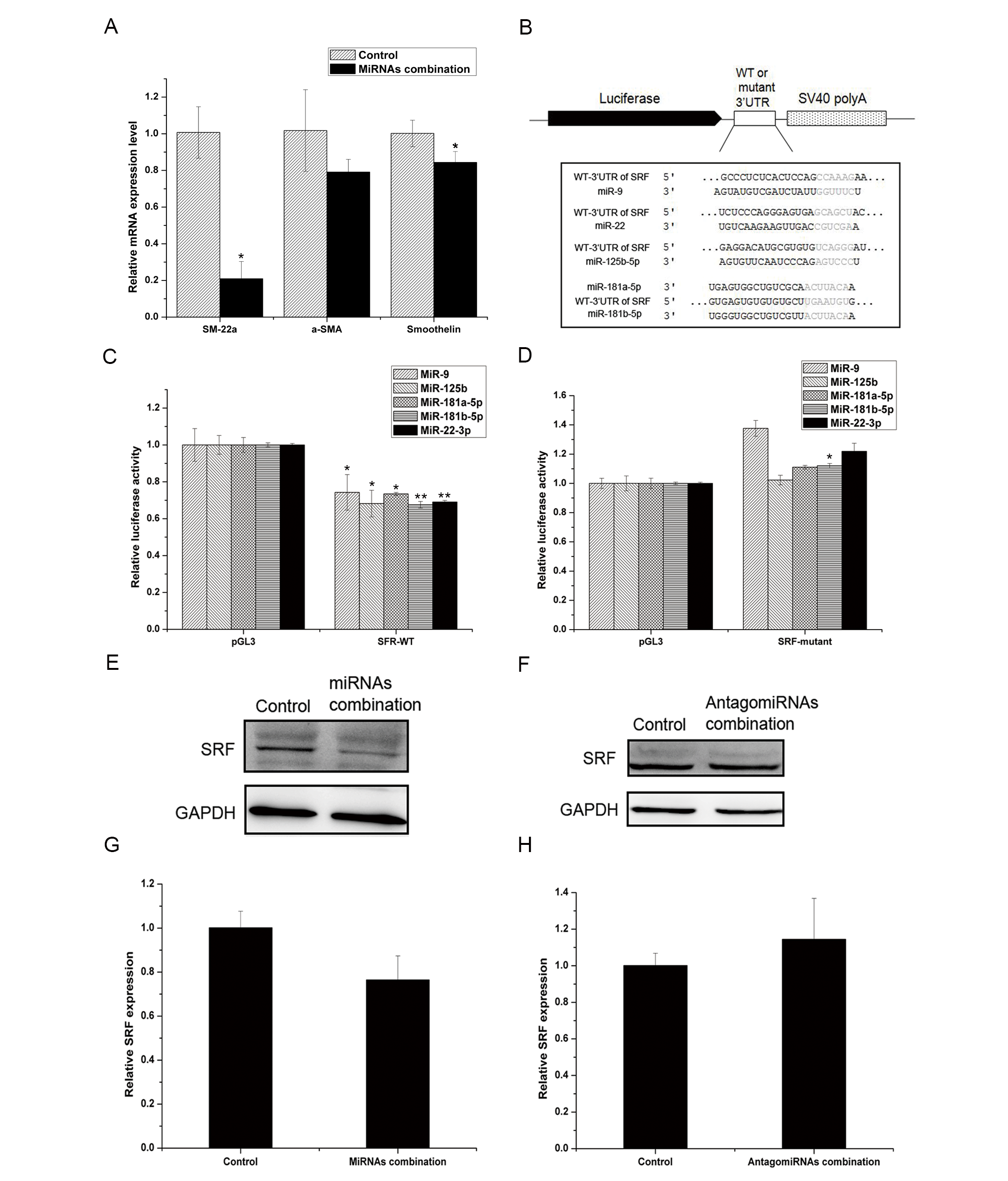

Supplement: S1 Fig — (A) Real-time PCR analysis of contractile marker gene expression (SM22α, α-SMA, and Smoothelin) in SMCs. (B) SRF-WT and SRF-mutant vectors co-transfected with five predicted miRNA oligos into HeLa cells. (C, D) Measurement of luciferase activity in HeLa cells. (E, F) Western blot analysis of SRF expression in SMCs co-transfected with five miRNAs (E) or antagomiRNAs (F). (G, H) Real-time PCR analysis of SRF expression in SMCs co-transfected with five miRNAs (G) or antagomiRNAs (H). Three independent experiments were performed for each condition, and data are presented as the mean ± SEM. * p<0.05 and ** p<0.01 versus control group. (TIF) [file pone.0152959.s001.tif]

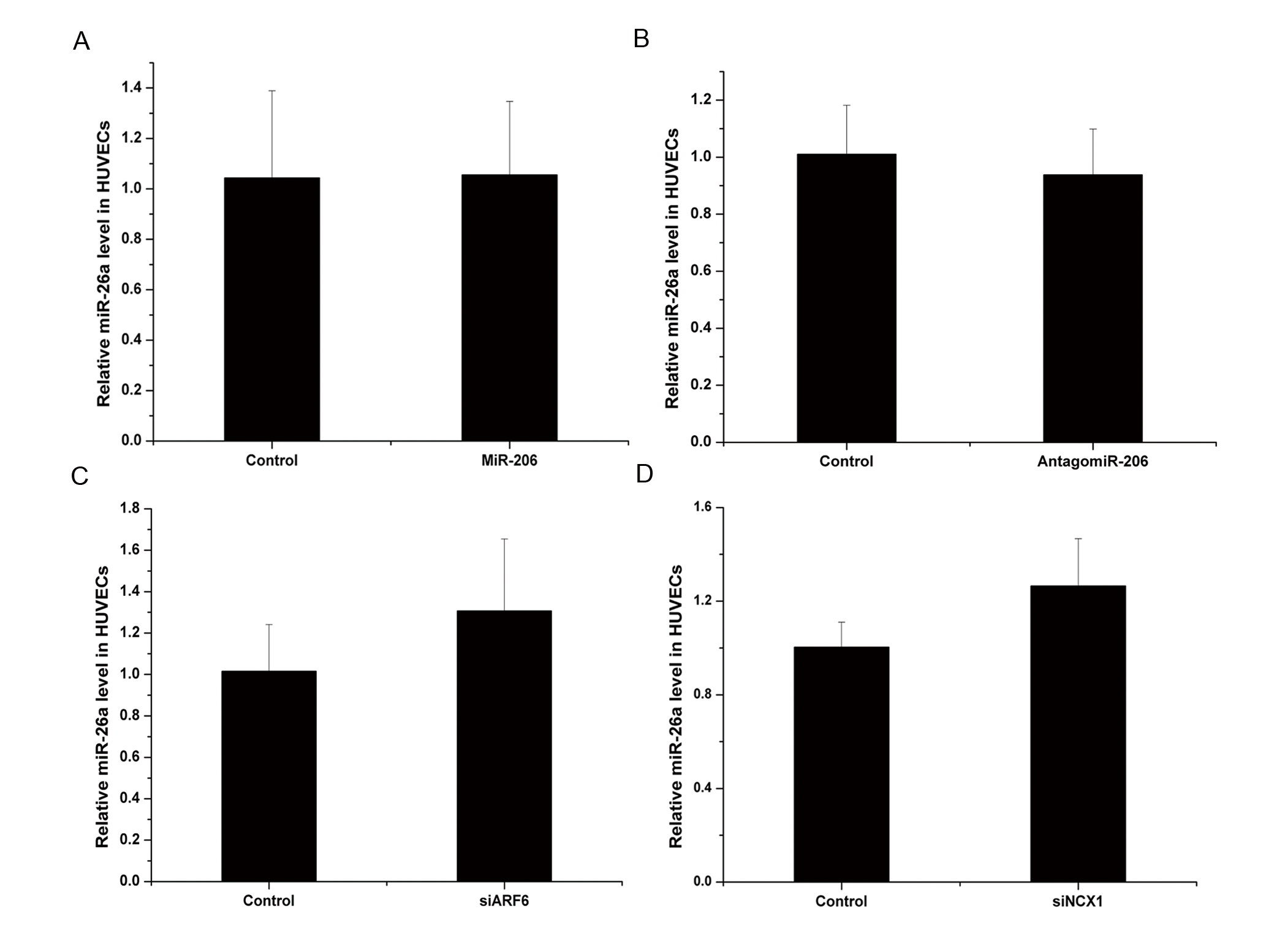

Supplement: S2 Fig — (A, B, C, D) Real-time PCR analysis of miR-26a expression in HUVECs transfected with miR-206 (A), antagomiR-206 (B), siARF6 (C) or siNCX1 (D). Three independent experiments were performed for each condition, and data are presented as the mean ± SEM. * p<0.05 and ** p<0.01 versus control group. (TIF) [file pone.0152959.s002.tif]
